# HRSA’s PEPFAR-Supported Resilient and Responsive Health Systems Initiative in Liberia

**DOI:** 10.5334/aogh.3122

**Published:** 2021-10-08

**Authors:** George Tidwell, Myat Htoo Razak

**Affiliations:** 1 Office of Global Health, Health Resources and Services Administration, US Department of Health and Human Services, LR

## Abstract

During the 2014-16 Ebola outbreak, Liberia suffered 2,544 deaths, including 8% of its doctors, nurses and midwives. The Government responded in 2016 with a seven–year Health Workforce Program (HWP), the goal of which was “the creation of a fit-for-purpose, motivated, and highly-skilled workforce.” The US Health Resources and Services Administration (HRSA) with the support from the US President’s Emergency Plan for AIDS Relief (PEPFAR), established Resilient and Responsive Health Systems project to assist Liberia in strengthening health workforce and health systems. As the HWP approaches its final year, progress the numbers and skills of physicians, nurses and midwives have improved, through national and global support, and there have been some improvements in the overall health system. Improving health for everyone in Liberia is an ongoing process that requires continuing support and collaboration from national and global partners including US government agencies, UN agencies, academic and training institutions, private foundations, regional networks, and especially the people of Liberia.

## Background

In 2016, the State Department Office of the Global AIDS Coordinator (S/GAC), which oversees the US President’s Emergency Plan for AIDS Relief (PEPFAR) invited the Health Resources and Services Administration (HRSA) to strengthen health workforce and health systems in four selected countries to become more resilient and responsive to health challenges like Ebola epidemics. Building on HRSA’s involvement with the PEPFAR Medical Education Partnership Initiative (MEPI) and Nursing Education Partnership Initiative (NEPI), these activities are supported through a PEPFAR Headquarters-based funding stream. Liberia is one of the four countries targeted for this support and is the subject of this commentary.

S/GAC allocates funding to individual countries based on the profile of the HIV epidemic and performance toward controlling the epidemic, and then approves the annual operating plans and performance targets developed by each country. Based on competencies, performance, country presence and other factors, individual USG agencies collaborate and propose agency-specific activities and associated funding as part of the operating plan submitted for S/GAC approval.

The development of this initiative represented a strong collaboration between the Office of the US Global AIDS Coordinator (OGAC) and HRSA, with both committed to revitalize a damaged and depleted health workforce in selected countries that had faced health crises threatening collapse of their health systems.

This initiative was required to emphasize strengthening health workforce and the health system to be resilient and responsive to above mentioned health challenges and priorities of the Country including HIV/AIDS, a global focus of PEPFAR.

During the 2014-16 Ebola outbreak, Liberia had 10,678 suspected, probable and confirmed cases, and 2,544 deaths, including 8% of its doctors, nurses and midwives.

The Minister of Health, Dr. Bernice Dahn, led development of a seven-year Health Workforce Program (HWP), which was unveiled in January 2016, the goal of which was “the creation of a fit-for-purpose, motivated, and highly-skilled workforce” – one of the building blocks for a resilient health system.

## Development of the Implementation Plan

Having led the Country through its Ebola crisis, the Ministry of Health set out to transform the health system, putting to good use and long-term service the wave of donor support in the wake of Ebola.

Throughout 2016, HRSA conducted two fact finding visits to Liberia which also involved substantive engagement with the Government of Liberia and other stakeholders. The resulting plan supports health workforce and systems, clinical staff as well as leadership and management, rural and urban. HRSA also led development of a team of experts from the West Africa College of Physicians to conduct accreditation readiness assessments of the primary clinical training sites recognizing that such accreditation would ultimately be necessary in support of the HWP and HRSA’s interventions.

A clear picture emerged of where PEPFAR support could have the greatest impact, and HRSA subsequently announced a grant opportunity for a Resilient and Responsive Health Systems Initiative (RRHS), and the announcement had specific requirements for each of the four countries.

HRSA collaborates with USAID, Centers for Disease Control and Prevention (CDC), Department of Defense (DOD) and other USG agencies in supporting Liberia in strengthening its health system. HRSA collaborates with the Global Fund to leverage this extensive investment, to avoid duplication, align objectives, and optimize impact. HRSA has also successfully advocated in extending the reach of Liberian stakeholders to other networks including Liberia becoming a member of AFREhealth, an interdisciplinary health professional grouping that seeks to work with Ministries of Health, training institutions and other stakeholders to improve the quality of health care in Africa through research, education and capacity building.

On January 1, 2017, HRSA awarded Brigham and Women’s Hospital (BWH) a five-year, $9.5 million cooperative agreement. BWH is leading a consortium of partners – New York University Rory Meyers College of Nursing, Yale School of Medicine, and Partners in Health – to support the successful implementation of the Liberia’s HWP. A similar consortium played an instrumental role in Rwanda’s innovative Human Resources for Health Program, which launched in 2012 and created a partnership between the Government of Rwanda and 26 academic institutions tasked with recruiting and deploying visiting faculty. The Rwanda program surpassed its workforce growth target, increasing the number of physician specialists from 150 in 2011 to more than 500 in 2018, and the number of nurses with advanced degrees from less than 800 to more than 5,000 [1]. Liberia’s HWP, inspired by Rwanda, similarly recruited visiting faculty to support the development of the Country’s health education system as local educators are produced and mentored.

The Yale School of Medicine is serving as the lead institutional partner for supporting and developing the internal medicine residency program in Liberia, as well as clinical training activities for students of the A.M. Dogliotti College of Medicine (AMD). Yale’s Global Health Leadership Institute (GHLI) is serving as the lead in a program supporting preclinical medical education and capacity building in basic science at AMD, working closely with existing faculty and in partnership with the MOH.

## Physician workforce projections at baseline, with HWP interventions depicting General Practitioner and Physician Specialist workforce

**Figure:**
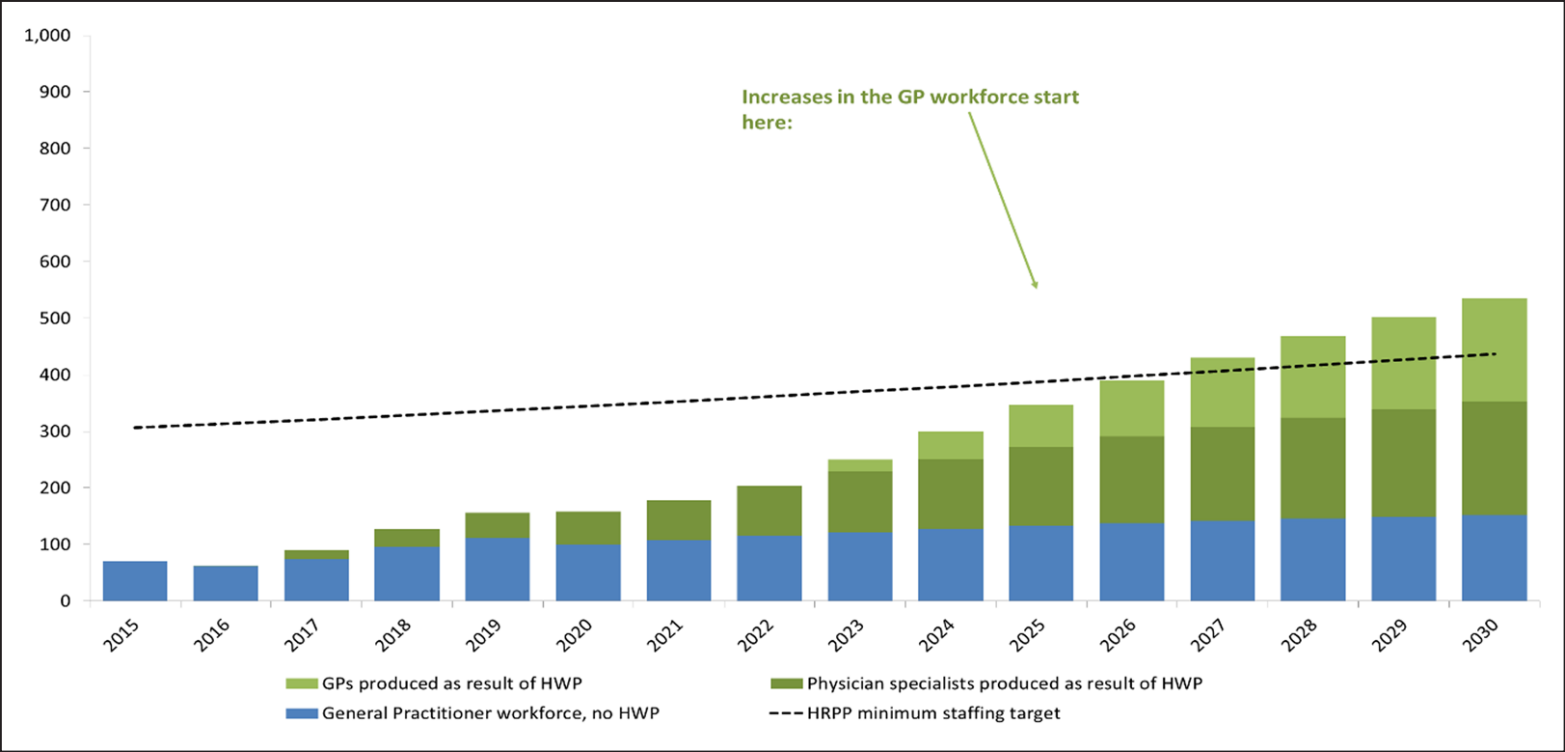


Yale GHLI is also supporting the health management component of the HWP to produce a cadre of effective health managers. Yale health management associates support a Health Management Certificate Training Program, now integrated into the University of Liberia and eligible for 12 credits in the Master of Public Health Program. The program supports mentoring and co-implementing QI projects in John F. Kennedy Memorial Medical Center, a large public tertiary hospital, and Redemption Hospital, a public hospital that does not charge for services and is the largest provider of secondary level services in the country. The projects created increased awareness in the clinics where HIV services are provided. Medical, nursing and midwifery training also occurs at these two hospitals.

NYU’s Rory Meyers College of Nursing (NYU) is the lead partner for nursing and midwifery for Liberia’s HWP. Working in partnership with the Ministry of Health (MOH) and the Liberia Board of Nursing and Midwifery (LBNM), NYU is strengthening education programs to train the next generation of Liberian faculty, nurses and midwives, including national dissemination of revised Nursing and Midwifery Curricula and introduction of a Master of Nursing and Midwifery Education program. NYU is also supporting development of continuous professional development for nurses and midwives, also in partnership with the MOH and the LBNM.

Partners in Health (PIH), working in rural areas of the Southeast Region of Liberia since 2014, engaged with the MOH and partners to establish a Rural Health Training program at J.J. Dossen Hospital and Pleebo Health Center, to train and mentor medical residents, rural service year physicians, and the local Tubman University nursing and midwifery students.

BWH collaborates with other US partners who are supporting other specialty medical training in pediatrics, obstetrics & gynecology, and surgery, all in support of the Liberia HWP.

HRSA, along with NIH, administered the PEPFAR-supported Medical Education Partnership Initiative (MEPI), and Stellenbosch University (SU) was among HRSA’s MEPI recipients from 2010-2015. SU has a robust eLearning system, and HRSA partnered with SU to implement an eLearning system at AMD in Liberia. Faculty and students eagerly embraced the new system.

**Figure:**
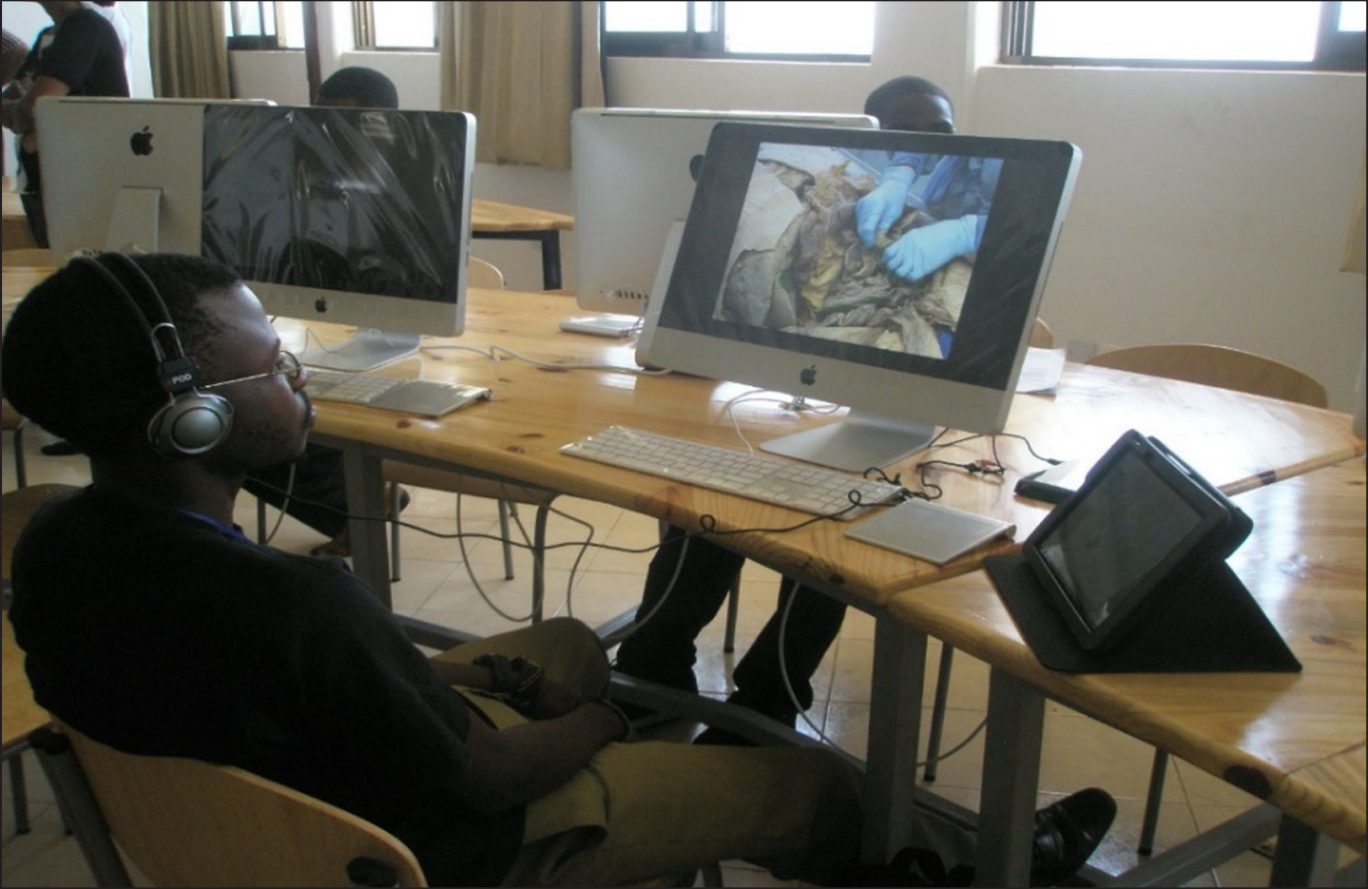


Dr. Dahn, who became the Vice President for Health Sciences at the University of Liberia after her Minister for Health position ended in 2017, introduced Lecturio, a high-quality digital medical education resource, to AMD, to leverage the existing eLearning platform. These strengthened resources have enabled medical education to continue during the COVID pandemic, which would have otherwise resulted in the suspension of medical education.

## Key Accomplishments

As the 5-year HWP approaches its final year (2021), the progresses have been seen especially in increasing numbers and skills of physicians, nurses and midwives as well as improving health system.

Forty-seven physicians have graduated within the first three and a half years of the five-year project, with an additional 82 physicians expected to graduate over the next three years. General physician training support to AMD included development and launch of a five-year strategic plan, introduction of internet services, establishment of an e-learning repository including lectures, training materials, and textbooks, and a Teaching and Learning workshop for 23 Liberian faculty, and assistance with on-boarding of visiting faculty supported by the World Bank.

The Internal medicine residency training program is strengthened through several approaches including recruitment and deployment of a few key core faculty and subspecialist faculty supported by the World Bank, USAID and HRSA, establishment of a teaching workshop, journal clubs and pedagogic innovations for faculty and senior residents and strengthening mentoring and supervision of Internal Medicine faculty in goal setting, progress monitoring, and student evaluation/competency assessment.

Hospital quality improvement projects have been implemented at JFK Medical Center and Redemption Hospital through RRHS-supported health management associates who also serve as faculty for a health management certificate training program. Over 10 quality improvement projects have been initiated. Some of the examples are:

Development of an automated patient registration and visit record at Redemption Hospital (RDH), overcoming inefficient registration including delays in locating medical records, and creation of duplicate records. Using an Access database, a Medical Record Management System was created, and staff at RDH can now search for medical records quickly and can also generate reports of numbers of inpatients and outpatient visits. Other examples included:

Redemption Hospital – Census reporting system and HMIS reporting system; central pharmacy inventory management system.

JFK Medical Center – Pathology laboratory reporting system; Infusion center registration system; central pharmacy inventory management system; Internal Medicine census reporting system; Internal Medicine renal disease registry.

The first cohort of 20 participants (from JFK Medical Center and RDH) in the Executive Certificate in Health Systems Leadership and Management (CHSLM) graduated from the University of Liberia College of Health Sciences School of Public Health. The second cohort includes individuals from the National AIDS Control Program and others.

**Figure:**
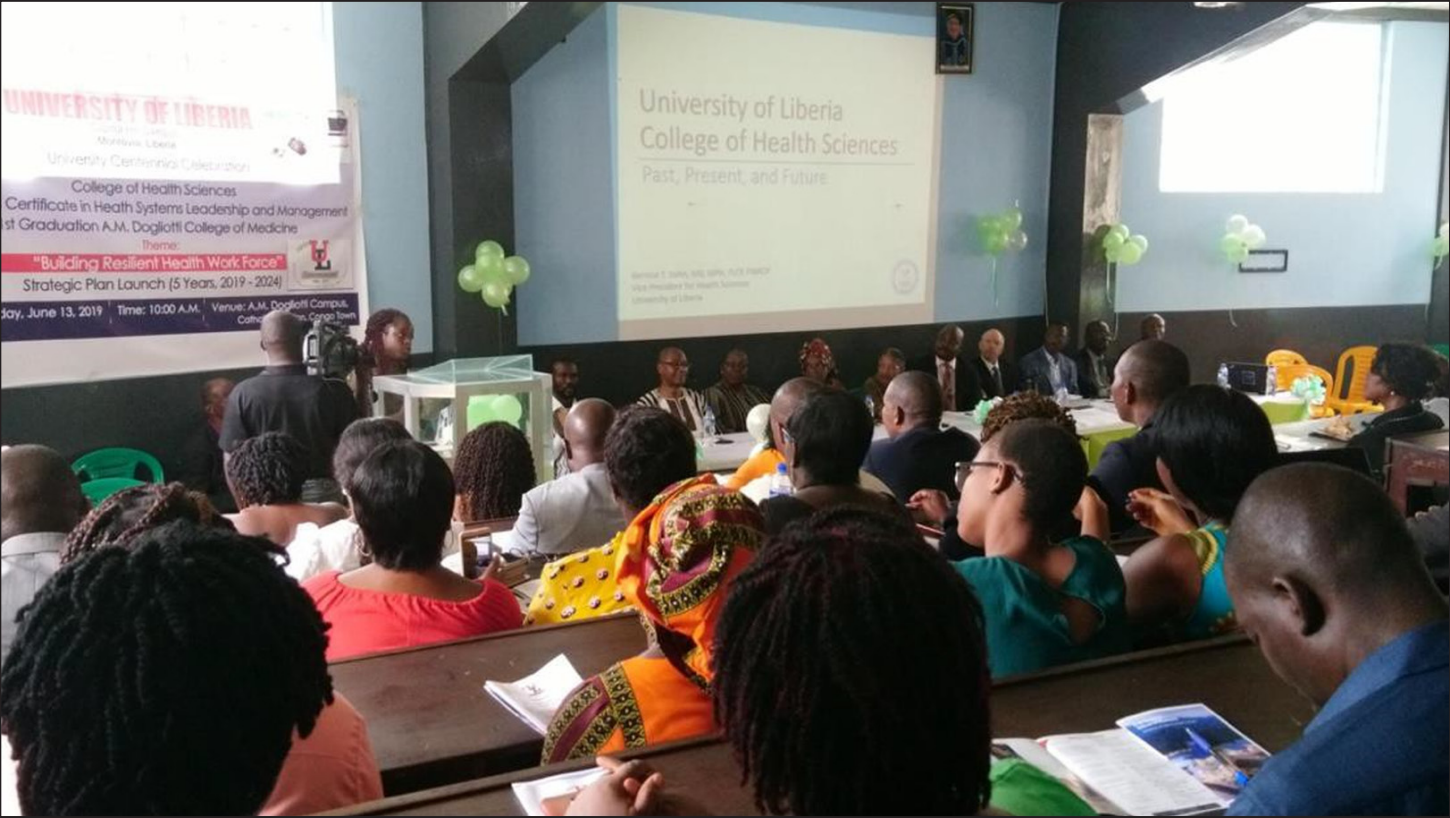


Midwifery education has been strengthened through comprehensive review, revision and dissemination of two national curricula, development and implementation of a Continuing Professional Development (CPD) module in Normal Birth Skills Training, training of 21 nurses and midwives from nine institutions in Montserrado County as trainers, development of the Midwifery component of a Master of Nursing and Midwifery Education in partnership with Mother Patern College of Health Sciences, and deployment of a full-time midwifery expert.

Support for nursing education strengthening included comprehensive review of national nursing curricula and facilitation of a stakeholders meeting, mentoring to the Registrar of the Liberia Nursing and Midwifery Board, Community Health Nursing teaching at rural Tubman University for 49 nursing students, bedside nurse mentor sessions at J.J. Dossen Hospital for 81 nurses, establishment of a conducive environment for on-site learning, by providing a medical library and supporting internet and electricity at JJ Dossen Hospital, providing simulation mannequins and textbooks for the nursing department, preparing Tubman University nursing students for licensing exams resulting in >90% pass rate, providing six CPD training sessions for nursing students and Ministry of Health nurses, and providing leadership and quality improvement training to 45 J.J. Dossen Hospital staff with ongoing QI projects.

As a component of strengthening rural health services, family medicine residency training included the establishment of a rural rotation site at J.J. Dossen Hospital, which hosted 11 family medicine residents in community health and primary care rotations for 27 months. Weekly continuing medical education meetings were held with general practitioners, residents and specialists at J.J. Dossen Hospital, resulting in a stronger community of family medicine doctors. A costed operational plan was developed for rural physician training. Infrastructure was added for residency training including an on-site call room and medical library. Physicians specialized in internal medicine, pediatrics, general surgery and obstetrics & gynecology were recruited to assist with training across the health workforce. These interventions are improving healthcare in rural and remote settings where the vast majority of Liberians live and access health care.

In recognition of the need to improve HIV/AIDS services and coverage, the project made some adjustments in collaboration with the Government of Liberia in 2019. A significant focus on HIV across the various training programs was made to increase the impact on beneficiaries of HIV services, with an emphasis to improve ART coverage and retention. All partners of the project collectively provided a comprehensive platform to deliver increased support for HIV services. HIV modalities were enhanced and emphasized across all training programs, and clinical rotations newly included outpatient HIV clinics.

In addition, the project is supporting training and quality improvement activities in Liberia’s two highest volume HIV sites substantially enhancing inpatient and outpatient services. These activities were informed by the human resources for health and HIV expertise of HRSA and its partners and were tailored for the context to assure maximum impact. Improvements in quality and coverage of HIV/AIDS services are experienced at the community level as the various cadres being supported reach their communities.

## Way Forward

The RRHS project in Liberia has played a key role in improving human resources for health and strengthening health systems in accordance with the Government of Liberia’s HWP.

Liberia is poised to achieve HIV epidemic control among the estimated 40,000 PLHIV in a few years, with a more resilient and responsive health workforce coupled with financial and technical support. Many important elements of effective HIV intervention programs such as community outreach, prevention, timely diagnosis, treatment and care approaches are similar to key elements of effective interventions of other communicable and non-communicable diseases. Therefore, through the support of PEPFAR, the RRHS project’s emphasis on HIV/AIDS, under HRSA’s umbrella approach of creating a more resilient and responsive health system, is far reaching for the Country.

Liberia’s Health Workforce Program will continue when HRSA’s current RRHS initiative concludes. There are a few areas in which global partners’ support and collaboration are likely to augment Liberia’s goal to improve health for everyone in the Country.

As an example, the PEPFAR DREAMS (Determined, Resilient, Empowered, AIDS-free, Mentored, and Safe) Partnership that supports interventions to address key factors that make girls and young women particularly vulnerable to HIV, would be very likely to be helpful to Liberia in effectively reducing underage pregnancy, HIV and high maternal mortality rate in Liberia.

Lessons learned from HRSA’s expertise in providing primary healthcare, HIV/AIDS treatment and care, rural health, and maternal and child health services, improving the health of underserved and vulnerable populations by strengthening health workforce and connecting skilled professionals with communities in need in the US, could be helpful to Liberia in addressing the Country’s priority health challenges.

In conclusion, Liberia has strengthened a diminished health workforce and weakened health system after the devastating Ebola outbreak, with national and global support. Improving health for everyone in Liberia is an ongoing process that requires continuing support and collaboration from national and global partners including US government agencies, UN agencies, academic and training institutions, private foundations, regional networks such as AFREhealth, and especially the people of Liberia. Such support and collaboration are essential for Liberia’s march towards a healthier country with active participation from people that strengthens sustainability and country ownership.
